# Combining rTMS and Task-Oriented Training in the Rehabilitation of the Arm after Stroke: A Pilot Randomized Controlled Trial

**DOI:** 10.1155/2013/539146

**Published:** 2013-12-03

**Authors:** Johanne Higgins, Lisa Koski, Haiqun Xie

**Affiliations:** ^1^Division of Experimental Medicine, McGill University, 1110 Pine Avenue West, Montreal, QC, Canada; ^2^School of Rehabilitation, Faculty of Medicine, Université de Montréal, C.P. 6128, Succursale Centre-Ville, Montreal, QC, Canada H3C 3J7; ^3^Centre for Interdisciplinary Research in Rehabilitation of Greater Montreal (CRIR), 2275, Avenue Laurier Est, Montréal, QC, Canada H2H 2N8; ^4^Division of Clinical Epidemiology, Research Institute of the McGill University Health Centre, 687, Pine Avenue West, Montreal, QC, Canada H3A 1A1; ^5^Division of Geriatrics, Research Institute of the McGill University Health Centre, 687 Pine Avenue West, Montreal, QC, Canada H3A 1A1; ^6^The Allan Memorial Institute, P2.142, McGill University Health Centre, 1025 Pine Avenue West, Montreal, QC, Canada H3A 1A1; ^7^Department of Neurology, Affiliated Foshan Hospital of Sun Yat-sen University, Foshan, Guangdong, China

## Abstract

*Introduction*. Repetitive transcranial magnetic stimulation (rTMS) is a promising technique for promoting rehabilitation of arm function after stroke. The feasibility and impact of rTMS as an adjunct to traditional task-oriented training to improve arm function have not yet been demonstrated. *Objective*. Evaluate the feasibility of a randomized controlled trial aimed at determining the efficacy of rTMS as an adjunct to task-oriented therapy in facilitating restoration of arm function after stroke. *Methods*. Stratified block-randomized controlled trial set in the general community. Eleven stroke persons with mild to severe arm deficits were recruited and randomized to receive 8 sessions of *real*-rTMS or *sham*-rTMS followed by ninety minutes of arm tasks designed to improve function. *Results*. Medium to large, statistically significant effect sizes (0.49 to 1.63) were observed in both groups on several measures of arm function at the postintervention evaluation. Three out of four subjects in the *real*-TMS condition showed increased levels of corticomotor excitability after the first stimulation session. *Conclusions*. Preliminary evidence suggests that an rTMS protocol potent enough to induce transient increases in cortical excitability of the lesioned hemisphere is feasible but did not show promising results as an adjunct to task-specific training. This trial is registration with Clinical Trials.gov NCT00850408.

## 1. Introduction

A burning question in the rehabilitation of stroke survivors is determining the most effective approach for improving recovery in persons who experience weakness of the arm and hand following a cerebrovascular accident (CVA). Two systematic reviews [1, 2] have suggested that intensity of stroke rehabilitation is an important factor associated with greater and faster improvement. However, there is growing pressure on rehabilitation professionals to increase effectiveness of treatments without increasing costs to health care systems. Repetitive transcranial magnetic stimulation (rTMS) is a promising noninvasive neuromodulatory intervention that aims to maximize recovery of function after stroke. Two distinct protocols have been employed: excitatory (high-frequency) stimulation of the lesioned hemisphere and inhibitory (low-frequency) stimulation of the unlesioned hemisphere. A few randomized controlled trials have confirmed that either low or high frequency rTMS has potential to improve motor function in the affected arm [3–7] and that rTMS might be used as an add-on to regular rehabilitation programs and drug therapies in acute stroke patients [6].

Recent studies on rehabilitation of arm function suggest that the effects of rTMS as a supplement to behavioral therapy may be influenced by time elapsed since stroke. In acute stroke, one trial compared 5 daily sessions of 1 Hz stimulations over the unaffected hemisphere versus 3 Hz over the affected hemisphere versus sham stimulation, showing enhanced recovery in both experimental groups [8]. A second trial concluded that real rTMS (3 Hz or 10 Hz over the affected hemisphere) produced greater improvement than *sham*-rTMS at one-year followup [9]. In contrast, two studies that included subjects in the chronic phase after stroke showed that high-frequency rTMS combined with constraint induced therapy was not superior to *sham*-rTMS combined with constraint induced therapy [10, 11]. Additional studies examined the combination of physical therapy with theta burst stimulation (TBS), a patterned variant of rTMS. In chronic stroke participants with mild to moderate deficits [12], motor recovery after 10 sessions of theta burst stimulation (TBS) in combination with physical therapy was no better than sham stimulation paired with physical therapy. In a study that included only persons with mild motor impairment, improvements in paretic hand performance were seen after inhibitory TBS of the unlesioned hemisphere, but not excitatory TBS of the lesioned hemisphere [13].

To summarize, the role of rTMS as a practical and feasible treatment modality for stroke rehabilitation has not yet been established. In particular, the benefits of combining rTMS with behavioral interventions are unknown for individuals in the chronic phase of stroke or for those with severe impairments that do not respond to traditional rehabilitative interventions. More research is needed to determine the most effective applications of rTMS and to determine realistic treatment intensity.

The present study, therefore, focused on a less intensive treatment regimen that required a more realistic time commitment for persons with chronic stroke, including those with severe arm and hand impairment, who are discharged from in-patient rehabilitation and required to travel to the site of intervention. rTMS was delivered immediately prior to task-specific training of the arm, on a schedule of twice a week over a period of four weeks. A protocol employing inhibitory (low-frequency) rTMS of the undamaged hemisphere was chosen for this study for its lower risk profile and because the duration of its neurophysiological effects on the brain may exceed that of high-frequency rTMS over the lesioned hemisphere [14]. This pilot study was designed to investigate preliminary support for the hypothesis that *real*-rTMS as an adjunct to task-specific training will result in greater improvement in arm function than *sham*-rTMS, among people with mild-to-severe hemiparesis at least three months after stroke. Concurrently, the feasibility of the participant recruitment, intervention delivery, and outcome measurement protocols were evaluated, as well as safety and adherence to the protocol.

## 2. Material and Methods

### 2.1. Design

This was a feasibility study of an observer-blinded stratified block-randomized controlled trial with *real*-rTMS (intervention group) versus *sham*-rTMS (control group) as an adjunct to task-specific training for arm function.

### 2.2. Subjects

This study was conducted at the McGill University Health Centre and approved by the institutional Research Ethics Board. Adults who had a first ischemic or hemorrhagic stroke at least 3 months previously that resulted in weakness of one arm were recruited through advertisements on hospital and community bulletin boards and by referral from hospital neurologists. All stroke diagnoses were confirmed by neuroimaging. Volunteers were excluded from participating if they met any of the following criteria: no residual motor impairment, complete paralysis of the hand and arm as measured by incapacity to produce the slightest voluntary contraction of any intrinsic hand muscle, previous cerebrovascular accident with persistent neurological sequelae, inability to provide informed consent, relative contraindications to rTMS (pacemaker, metal in the head, personal history of seizures, and taking medications known to lower the seizure threshold), other neurological disorders or major medical incapacity. Lesion location was confirmed by review of the clinical record. Eleven participants were recruited to the study over an 18-month period.

### 2.3. Intervention

Subjects in both groups participated in a four-week, twice weekly functional enhancement program that included task-oriented training aimed at improving functional use of the affected arm. One group received *real*-rTMS at the start of each visit, immediately before a session of task-oriented training. The other group received *sham*-rTMS before the training sessions, using a coil that mimics the look and sound of the real coil, ensuring blinding of participants as to group assignment. When asked to guess which group they had been assigned to, one person in each group guessed correctly and one person in the *real*-rTMS group guessed differently at the two evaluation time points. rTMS was administered using a Magstim Rapid2 stimulator with an air-cooled figure-8 coil placed over the optimal spot for stimulating the first dorsal interosseous (FDI) muscle, important for grasping and pinching actions, the basis for most functional arm and hand movements. A frameless stereotaxic system (Rogue Research, Canada) was used to ensure reliable placement of the coil across all stimulation sessions and throughout the collection of neurophysiological data (see below). Stimulation parameters were selected based on those demonstrated in previous work to be effective for modifying corticomotor excitability [15] and improving poststroke motor function [3–5].

*rTMS Intervention. *The center of the figure-8 coil was positioned over the motor cortex of the unaffected hemisphere and oriented perpendicular to the central sulcus for optimal stimulation of the underlying tissue. 1200 pulses were delivered at a frequency of 1 Hz, at an intensity of 110% of the motor threshold [13, 16] as established at baseline. *Sham-rTMS *procedures were identical to those used for *real*-rTMS except that a placebo coil was used [17].

*Task-Oriented Training*. The program consisted of a structured series of 90-minute sessions delivered by a trained occupational therapist (JH) unaware of the participants' group allocation. The content focused on the repetition and relearning of goal-oriented tasks and activities performed with the affected arm and hand at a level that was challenging but within each individual's potential. The sessions were individually tailored to the participants' goals, life roles, and level of functional ability. Activities included reaching, grasping, and manipulation of functional objects. Depending on the individual's level of ability, examples of activities may have included opening jars, stacking objects, grasping and eating finger foods, handwriting, and computer mouse manipulating and typing. The therapist matched the activities to functional level and implemented any needed techniques for stabilization of the arm or for facilitation of the movement. Each task was repeated on average 10–15 times for a duration of approximately 10 to 15 minutes so that about nine different tasks were practiced at each session. A record of the activities performed (nature, duration, and difficulty) was kept at each session. In addition, subjects were given homework assignments consisting of activities aimed at increasing the use of the affected arm in daily life.

### 2.4. Outcome Measures

Evaluations were conducted by trained rehabilitation professionals upon entry in the study (median 39 months after stroke-range 17 to 301), upon completion of the intervention (mean 4 days after completion), and one month later (mean 34 days). Evaluators for postintervention and follow-up evaluations were unaware of the participants' group assignment. Measures of capacity, motor impairment, and motor excitability were obtained before and at all evaluation time points after the intervention. Measures of motor excitability were also obtained immediately after a session of *real*- or *sham*-rTMS administered at the baseline evaluation.

#### 2.4.1. Measures of Motor Impairment and Capacity

The Box and Block Test (BBT) [18] was selected as the primary measure of arm function. It is a brief [19, 20] performance-based estimate of unilateral gross manual dexterity where subjects must move as many blocks as possible from one box to another in 60 seconds. Other measures included the Motor Function Test (WMFT) [21–23], the Motor Activity Log-14 (MAL-14) [24], grip strength, and pinch strength [25]. The Stroke Impact Scale (SIS-16) [26] and the Participation component of the SIS questionnaire were also administered.

#### 2.4.2. Measure of Motor Excitability

It was hypothesized that rTMS might improve functional outcome by promoting increased neural activity during motor skills training. To test this hypothesis, the effect of rTMS on corticomotor excitability was measured before and after the first rTMS session, administered prior to any task-oriented training. Corticomotor excitability was measured using motor evoked potentials (MEPs). Electromyographic signals were acquired using surface electrodes applied in a belly-tendon montage over the FDI muscles of the left and right hands. Signals were filtered between 1 and 1000 Hz, amplified, and digitized at a sampling rate of 20 kHz. Data were visually displayed and stored for later analysis in samples of 170 ms to include the window of time from 100 ms before TMS to 70 ms after TMS. Single TMS pulses were delivered using a Magstim 200 stimulator with a 70 mm figure-8 coil, with an interpulse interval of 5–10 seconds. The coil was oriented perpendicular to the central sulcus for optimal stimulation of the underlying cortical neurons [27]. The “hotspot” corresponded to the stimulation site for which the largest MEPs were obtained in the contralateral FDI. Resting motor threshold was determined as the minimum TMS stimulator intensity capable of eliciting a motor response ≥50 *μ*V in the muscle in at least 3 of 6 consecutive trials [28], with confirmation of the best stimulation site by testing 4–8 locations within 1 cm of the identified hotspot at 1% below threshold intensity. Recruitment curves were obtained for both the ipsilesional and contralesional motor cortex. Ten MEPs were obtained at each of a series of stimulus intensities ranging from just below threshold (95%) to 100% of maximum stimulator output, increasing by 5% intervals.

### 2.5. Randomization

Subjects were stratified according to two levels of arm deficit as measured by the Chedoke-McMaster Stroke Assessment hand subscale [29]: mild-moderate if they had a score of 5 or 6 and severe if they had a score of 3 or 4 out of a possible 7. The sequence of random assignments was computer generated in randomly ordered block sizes of two and four for each stratum. After consent and baseline assessment were obtained, evaluators stratified subjects and confirmed their treatment assignment with the person in charge of the randomization list who was not involved with the study.

### 2.6. Analyses

Analyses were done with SAS statistical software (version 9.1) and performed by an investigator blind to treatment allocation following intention-to-treat protocol. Change scores were calculated comparing baseline to postintervention (after the one-month series intervention), and baseline to the 4-week followup. Effect sizes and 95% confidence intervals were calculated for both groups for the BBT, as well as for the secondary outcome measures (grip strength, pinch strength, the WMFT, the MAL, and the SIS total score and SIS participation). To compare the MEP amplitudes before versus after stimulation at the baseline evaluation, the MEP amplitudes obtained at each intensity were averaged and the responsiveness of each subject's ipsilesional motor cortex to rTMS (real or sham) was assessed using a *t*-test, paired by stimulus intensity.

## 3. Results

### 3.1. Recruitment

**Figure 1:**
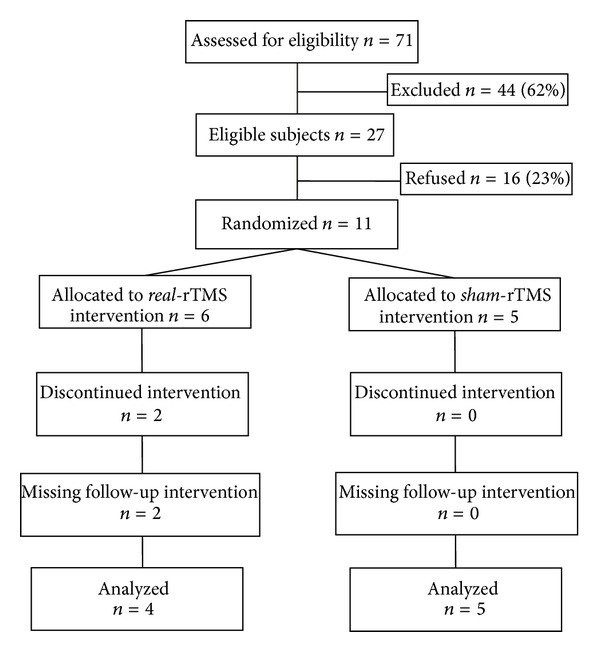
Flow of subjects through the trial according to the CONSORT statement (rTMS, repetitive Transcranial Magnetic Stimulation).

The charts of seventy-one persons were initially screened for eligibility into the study (Figure 1 illustrates the flow of participants according to the CONSORT statement) [30]. Of those screened, 22% (*n* = 16) refused to take part in the study. Most did not give specific reasons. Some mentioned the time commitment involved and travelling to the research site as reasons for not participating. Sixty-two percent (*n* = 44) did not meet the eligibility criteria. Common reasons for exclusion were related to medical conditions that were incompatible with TMS (*n* = 6), absence of any voluntary movement in the hand (*n* = 9). Eight persons confirmed that their arm function was never affected by the stroke or they experienced a full recovery. Eleven persons were recruited and randomized.

### 3.2. Participant Flow and Characteristics of Participants

Of the eleven subjects recruited, two subjects withdrew from the study before the postintervention evaluation (Figure 1). One withdrew following an adverse event during but not related to the intervention session. The subject experienced an important drop in blood pressure, having taken the wrong dose of medication on the morning of the treatment. He had completed five intervention sessions. The second participant withdrew following four intervention sessions. She had sustained a fall at home and was no longer able to travel for the study.

Subjects' characteristics are presented in Table 1. Subjects randomized to the *real*-rTMS groups were older (70 versus 60 for the *sham*-rTMS) and had had their stroke for a longer period before entering the study. The number of women was approximately the same for both groups and all participants were right-handed. Persons allocated to the *real*-rTMS group obtained a slightly higher score on the BBT than persons allocated to the control group (27.5 versus 22. blocks, resp.).

### 3.3. Intervention and Subject Compliance

Of the nine subjects who completed the intervention protocol, all attended the twelve treatment sessions as scheduled and complete data were collected at the three time points (baseline, postintervention, and followup) on all outcome measures for these participants. No subjects reported experiencing headaches or any sort of discomfort following the rTMS or the behavioral intervention.

### 3.4. Outcome Measures-Motor Impairment and Capacity

Scores for both groups on motor impairment and capacity measures are presented in Table 2. On average, subjects in both groups increased the number of blocks transferred by four (4) at the postintervention evaluation and this difference decreased by 1 block at the follow-up evaluation on the main outcome measure, the BBT.

Table 3 presents within- and between-group effect sizes for all measures of impairment and capacity. No effect of the *real*-rTMS intervention in comparison to the *sham*-rTMS was found at the postintervention or at the follow-up evaluations on the BBT. Despite not reaching statistical significance, trends for small to large between-group effect sizes (0.13 and 0.67) for the functional and times scales of the WMFT and for the quality part of the MAL, respectively, were observed at the postintervention evaluation. Medium effect sizes (0.34 and 0.46) were also observed for the latter measures at the follow-up intervention.

Upon examination of the effect sizes obtained from within group analyses, participants in both randomization groups exhibited improvements on the BBT, in pinch strength, on the WMFT (functional score), and the on the SIS at the postintervention evaluation. In addition, the group randomized to the *sham*-rTMS intervention exhibited improvement on the SIS participation, as well as on the Quality of Movement score of the MAL. Gains obtained on the functional score of the WMFT were not only maintained at the follow-up evaluation, but the effects sizes are larger for both groups.

For the *real-TMS *group, gains in pinch strength were maintained at followup, but gains that had been made on the BBT and the SIS were lost. For the *sham-TMS *group gains in pinch strength and on the SIS were lost at the follow-up evaluation and although the effect sizes are significant, they decreased for the BBT, the SIS participation, and the MAL.

### 3.5. Outcome Measures-Motor Excitability

Comparisons of the recruitment curves in the lesioned hemisphere before and after rTMS at the baseline evaluation revealed that they were significantly higher after the *real*-rTMS than after the *sham*-rTMS for three out of the four participants randomized to the *real-*TMS group (*P* < 0.01), providing evidence for the potency or rTMS to induce transient increases in cortical excitability of the lesioned cerebral hemisphere. For participants randomized to the *sham-*TMS group, none showed differences in excitability between the pre- and post-*sham-*rTMS invention performed at the baseline visit.

## 4. Discussion

This pilot study aimed to evaluate the effects of rTMS combined with task-oriented training in hemiparetic persons in the postacute stages of stroke recovery. Specifically, we looked at recruitment rate, feasibility of the intervention, including intensity and duration of treatment, and acceptability and feasibility of assessment schedule. An intention-to-treat analysis was also performed to determine the effects of rTMS combined with task-oriented training on arm function.

### 4.1. Recruitment Rate and Loss to Followup

Recruitment rate was disappointing, that is, 11 persons over an 18-month period. Over 50% of the persons screened for eligibility were not eligible to participate. Although we had specific and relatively stringent criteria for inclusion into the study, these criteria do not differ significantly from those used in similar studies [3, 5]. A common reason for noneligibility was level of arm function. It was either too low or too high. A possible reason for this is that most of the referrals to our study came from previous studies led by our group of researchers on persons with stroke. These studies, however, did not have exclusion criteria based on level of arm function and a good number of their participants did not meet our criteria based on information collected during their participation and thus were not approached or further screened. Those that met the eligibility criteria were contacted through mailings from contact information available in the medical records and this may not be a very effective method [31]. In fact, 35% of persons who participated in other research projects and had expressed a willingness to participate in future studies either refused to partake in this one or did not reply to our invitation. Unfortunately, reasons for unwillingness to participate were not expressed.

Another recruitment strategy was posting advertisements on bulletin boards and on our laboratory website. Only a handful of persons called to enquire about the study and of those who did, none were eligible to participate. Brochures explaining the study were distributed in neurologists' offices and to health professionals working with persons with stroke. They were also distributed in two rehabilitation centers. Professionals referred most of the persons screened for eligibility to us and they referred all of the eleven participants in this study. Other common, unavoidable reasons limiting recruitment were presence of comorbidities, some of which were simply incompatible with the use of TMS. Two subjects initially randomized did not complete the study as planned. Although the reason for their withdrawal was not directly related to the study itself, it is reasonable to assume that health problems prevented their travelling to the research site and tolerating over two hours of therapy two times per week.

### 4.2. Feasibility of the Intervention

The nine participants that completed the study attended all eight treatment sessions and evaluations as per schedule and did not experience adverse events related to the rTMS stimulations or the behavioral intervention. Hence, administering low-frequency rTMS for a period of twenty minutes two times a week followed by a ninety-minute task-oriented intervention is both feasible and acceptable in terms of subject safety and tolerance.

### 4.3. Intention-to-Treat Analyses

This study provided evidence that a twenty-minute session of low-frequency rTMS with an intensity equal to 110% of the motor threshold applied to the unlesioned side has a transient effect on excitability of the corticomotor pathways in patients of an advanced age with chronic stroke. This implies that the uninjured hemisphere is receptive to modulation in the short term. Although rTMS had transient effects on the brain, our study failed to demonstrate a significant effect of rTMS as an adjunct to task-oriented therapy. The effect size obtained from the comparison of *real*- versus *sham*-rTMS groups on our primary outcome measure was less than 0.01. While acknowledging that this estimate is limited by a small sample size, we cannot fail to note that the preliminary evidence is not promising in terms of demonstrating the effectiveness of low-frequency rTMS protocol as an adjunct to task-oriented therapy among people with hemiparesis at least three months after stroke.

Our results are in accordance with those of three randomized trials that evaluated the effects of high-frequency rTMS over the lesioned hemisphere [10, 11] or low frequency rTMS over the nonlesioned hemisphere [32] as an adjuvant to behavioral therapy. They were unable to establish a difference between subjects randomized to the *real*-rTMS and those randomized to the *sham*-rTMS group.

On the other hand, a study that investigated the long-term effects of rTMS (10 daily sessions of 1 Hz rTMS over the intact motor cortex) as an adjuvant to physical therapy in chronic stroke patients with mild motor disabilities found greater behavioral and neurophysiologic outcomes after *real*-rTMS when administered before physical therapy treatments [33]. Our study, however, included persons whose arm and hand were severely affected with only the most minimal demonstrable muscle control, who do not benefit from traditional therapy and who would thus be the best candidates for testing more novel approaches.

Another trial also looked at the effects of low-frequency rTMS (10 daily sessions of 1 Hz rTMS) to the contralesional motor cortex at an early stage of mild to severe hemiparesis. There were significant improvements in performance on the Jebsen-Taylor test pinch force in the *real*-rTMS group, but not in the *sham*-rTMS group. However, both groups improved significantly on the arm subscale of the Fugl-Meyer and on the Modified Rankin Scale [34].

We chose to implement a more realistic treatment regimen, one that would resemble a protocol that could be offered on an outpatient basis for persons with chronic stroke. Although its potency to improve function was not demonstrated, the intervention, as delivered, proved to be acceptable for persons who have been discharged home and who travel to the treatment site.

Besides our limited sample size, there may be several reasons explaining failure to detect an rTMS effect. The dose and intensity of the rTMS, although based on empirical data, may not have been optimal. Indeed studies differ greatly on this aspect and one cannot draw conclusions at this time as to the best approach to promote lasting changes in corticomotor pathways. The exact parameters with which to administer the rTMS are not well established and effects may vary greatly depending on interindividual variability such as the site and size of the lesion and the severity of the impairment, as well as intraindividual variability [35, 36]. It has also been suggested that for some participants, the therapy itself may increase outcomes to their ceiling and therefore make detection of an effect of rTMS impossible [37].

Examination of within group effect sizes indicated that our task-oriented arm intervention had an effect on arm function. Participants in both groups exhibited improvement on the BBT and effect sizes were medium to large for the *real*-rTMS and *sham*-rTMS groups, respectively. Two persons showed clinically significant changes on the BBT at the postintervention evaluation (7 blocks), one in each of the groups. Interestingly, the larger effect sizes were observed on the WMTF for both groups. One possible explanation for this observation is that tasks evaluated with this test resemble tasks that were practiced during the behavioral intervention. The task-oriented therapy failed to show consistent lasting changes at the follow-up evaluation. One cannot rule out that the dose or intensity, as it was delivered, was not optimal and the impact of adding rTMS could therefore not be detected.

## 5. Conclusion

Preliminary evidence from this study suggests that an rTMS protocol that was potent enough to induce transient increases in cortical excitability of the lesioned cerebral hemisphere in persons with stroke, nevertheless, did not show promising results when used as an adjunct to task-specific training aimed at improving arm function. Our results suggest that task-oriented therapy itself can have a beneficial effect on arm function even in older individuals with chronic stroke, at least in the short term.

At this time, studies looking at the adjunctive role of rTMS to arm therapy after stroke vary greatly in (1) the type of subjects they include (time after stroke, level of severity, site, and size of lesion), (2) the parameters of rTMS administration, and (3) the type of behavioral therapy (robotic, constraint induced, and task-oriented). It is therefore difficult at this time to draw conclusions regarding the clinical indications for the combined use of rTMS modality and behavioral therapy.

Further studies should investigate the influence of interindividual characteristics such as the size and site of lesion on the response to rTMS as well as behavioral therapy, as the specific parameter with which to administer both types of treatment may vary greatly between individuals. An in-depth understanding of the mechanisms of action of each approach is essential to guide the development of these combined treatment approaches.

## Figures and Tables

**Table 1:** Baseline characteristics of study subjects who completed the study.

Characteristics	rTMS group	*Sham* group
	*n* = 4	*n* = 5
Age in years, mean (SD) range	74 (8) 66–83	60 (11) 47–76
Gender, men/women	3 (75)/1 (25)	3 (60)/2 (40)
Side of hemiplegia, left/right	1 (25)/3 (75)	1 (25)/4 (75)
Number of months after stroke at baseline, mean (SD) range	134 (125) 32–315	95 (117) 18–301
Number of comorbid conditions, 0/1-2/3-4/>4	1/0/3/0	1/3/0/1
Right handedness	4	5

SD: standard deviation.

**Table 2:** Scores on measures of motor impairment and capacity at baseline, postintervention, and follow-up evaluations.

Test	*sham*-rTMS		*real*-rTMS	
	Mean (SD) baseline	Mean (SD) postintervention	Mean (SD) baseline	Mean (SD) postintervention
BBT affected (number of blocks)	22.8 (20.2)	27.0 (20.2)	27.5 (18.9)	31.5 (19.2)
Grip strength (kg)	19.3 (17.0)	18.8 (12.8)	14.2 (15.0)	15.1 (12.7)
Pinch strength kg (kg)	5.3 (2.5)	5.6 (2.4)	5.5 (4.6)	6.0 (3.6)
SIS (/80)	66.2 (16.4)	69.6 (15.1)	66.5 (10.3)	71.2 (4.6)
SIS participation (/40)	31.8 (9.5)	35.0 (7.3)	28.2 (10.1)	28.5 (10.8)
WMFT functional scale (/75)	53.6 (32.2)	63.8 (27.0)	69.5 (27.8)	75.0 (24.8)
WMFT times tasks (sec.)	16.4 (22.2)	16.4 (27.5)	4.5 (6.5)	3.9 (2.1)
MAL quality of movement (/70)	24.8 (26.4)	37.4 (27.9)	35.8 (24.8)	31.2 (17.6)

FU: followup. ES: effect size. CI: confidence interval. SD: standard deviation. BBT: Box and Blocks Test. SIS: Stroke Impact Scale. WMFT: Wolf Motor Function Test. sec: seconds. MAL: Motor Activity Log.

**Table 3:** Within and between-group comparisons for measures of motor impairment and capacity at baseline, postintervention, and follow-up evaluations.

TEST	Within group *real*-rTMS Mean age = 70		Within group *sham*-rTMS Mean age = 60		Between group	
	Post-	FU	Post-	FU	Postintervention	FU
	ES (95% CI)	ES (95% CI)	ES (95% CI)	ES (95% CI)	ES (95% CI)	ES (95% CI)
BBT affected	0.49* (−0.08–1.06)	0.05 (−0.72–0.8)	0.98* (0.28–1.69)	0.37* (0.08–0.66)	0.00 (−0.51–0.51)	0.02 (−0.76–0.81)
Grip strength	0.15 (−0.24–0.53)	0.24 (−0.16–0.61)	−0.10 (−0.23–0.03)	−0.30 (−0.61–0.01)	−0.09 (−0.48−0.29)	−0.18 (−0.59–0.22)
Pinch strength	0.51* (0.09–0.94)	0.66* (0.13–1.19)	0.54* (0.12–0.97)	0.05 (−0.17–0.27)	−0.01 (−0.28–0.26)	−0.23 (−0.52–0.07)
SIS	0.78* (0.08–1.49)	−0.06 (−0.48–0.36)	0.93* (0.26–1.60)	0.15 (−0.11–0.41)	−0.38 (−1.00–0.24)	0.08 (−0.40–0.56)
SIS participation	0.08 (−0.23–0.38)	−0.05 (−1.25–1.15)	0.71* (0.07–1.36)	0.38* (−0.06–0.82)	0.35 (−0.22–0.93)	0.22 (−1.03–1.47)
WMFT functional	1.18* (0.23–2.14)	0.93* (0.17–1.69)	1.63* (0.50–2.76)	1.06* (0.30–1.81)	0.13 (−0.20–0.47)	0.34 (−0.13–0.80)
WMFT time	−0.72 (−1.33–0.12)	0.41 (0.05–0.77)	0.00 (−1.09–1.09)	−0.41 (−1.55–0.70)	0.24 (−0.88–1.36)	−0.82 (−2.01–0.37)
MAL quality	−0.30 (−0.96–0.35)	0.02 (−0.86–0.91)	1.20* (0.30–2.10)	1.46* (0.41–2.51)	0.67 (−0.11–1.45)	0.46 (−0.52–1.45)

FU: followup. ES: effect size. SD: standard deviation. BBT: Box and Blocks Test. SIS: Stroke Impact Scale. WMFT: Wolf Motor Function Test. MAL: Motor Activity Log.

*Indicates statistically significant ES.

## References

[1] Kwakkel G, Wagenaar RC, Koelman TW, Lankhorst GJ, Koetsier JC (1997). Effects of intensity of rehabilitation after stroke: a research synthesis. *Stroke*.

[2] Langhorne P, Wagenaar R, Partridge C (1996). Physiotherapy after stroke: more is better?. *Physiotherapy Research International*.

[3] Mansur CG, Fregni F, Boggio PS (2005). A sham stimulation-controlled trial of rTMS of the unaffected hemisphere in stroke patients. *Neurology*.

[4] Takeuchi N, Chuma T, Matsuo Y, Watanabe I, Ikoma K (2005). Repetitive transcranial magnetic stimulation of contralesional primary motor cortex improves hand function after stroke. *Stroke*.

[5] Fregni F, Boggio PS, Valle AC (2006). A sham-controlled trial of a 5-day course of repetitive transcranial magnetic stimulation of the unaffected hemisphere in stroke patients. *Stroke*.

[6] Khedr EM, Ahmed MA, Fathy N, Rothwell JC (2005). Therapeutic trial of repetitive transcranial magnetic stimulation after acute ischemic stroke. *Neurology*.

[7] Kim Y-H, You SH, Ko M-H (2006). Repetitive transcranial magnetic stimulation-induced corticomotor excitability and associated motor skill acquisition in chronic stroke. *Stroke*.

[8] Khedr EM, Abdel-Fadeil MR, Farghali A, Qaid M (2009). Role of 1 and 3 Hz repetitive transcranial magnetic stimulation on motor function recovery after acute ischaemic stroke. *European Journal of Neurology*.

[9] Khedr EM, Etraby AE, Hemeda M, Nasef AM, Razek AAE (2010). Long-term effect of repetitive transcranial magnetic stimulation on motor function recovery after acute ischemic stroke. *Acta Neurologica Scandinavica*.

[10] Malcolm MP, Triggs WJ, Light KE (2007). Repetitive transcranial magnetic stimulation as an adjunct to constraint-induced therapy: an exploratory randomized controlled trial. *American Journal of Physical Medicine and Rehabilitation*.

[11] Richards L, Rothi LJG, Davis S, Wu SS, Nadeau SE (2006). Limited dose response to Constraint-Induced Movement Therapy in patients with chronic stroke. *Clinical Rehabilitation*.

[12] Talelli P, Wallace A, Dileone M (2012). Theta burst stimulation in the rehabilitation of the upper limb: a semirandomized, placebo- controlled trial in chronic stroke patients. *Neurorehabilitation and Neural Repair*.

[13] Talelli P, Greenwood RJ, Rothwell JC (2007). Exploring Theta Burst Stimulation as an intervention to improve motor recovery in chronic stroke. *Clinical Neurophysiology*.

[14] Fitzgerald PB, Brown TL, Daskalakis ZJ, Chen R, Kulkarni J (2002). Intensity-dependent effects of 1 Hz rTMS on human corticospinal excitability. *Clinical Neurophysiology*.

[15] Fitzgerald PB, Fountain S, Daskalakis ZJ (2006). A comprehensive review of the effects of rTMS on motor cortical excitability and inhibition. *Clinical Neurophysiology*.

[16] Lang N, Harms J, Weyh T (2006). Stimulus intensity and coil characteristics influence the efficacy of rTMS to suppress cortical excitability. *Clinical Neurophysiology*.

[17] Lisanby SH, Gutman D, Luber B, Schroeder C, Sackeim HA (2001). Sham TMS: intracerebral measurement of the induced electrical field and the induction of motor-evoked potentials. *Biological Psychiatry*.

[18] Cromwell FS (1965). *Occupational Therapist's Manual for Basic Skill Assessment; Primary Prevocational Evaluation*.

[19] Desrosiers J, Bravo G, Hebert R, Dutil E, Mercier L (1994). Validation of the Box and Block Test as a measure of dexterity of elderly people: reliability, validity, and norms studies. *Archives of Physical Medicine and Rehabilitation*.

[20] Mathiowetz V, Volland G, Kashman N, Weber K (1985). Adult norms for the box and block test of manual dexterity. *The American Journal of Occupational Therapy*.

[21] Morris DM, Uswatte G, Crago JE, Cook EW, Taub E (2001). The reliability of the wolf motor function test for assessing upper extremity function after stroke. *Archives of Physical Medicine and Rehabilitation*.

[22] Wolf SL, Catlin PA, Ellis M, Archer AL, Morgan B, Piacentino A (2001). Assessing wolf motor function test as outcome measure for research in patients after stroke. *Stroke*.

[23] Wolf SL, Thompson PA, Morris DM (2005). The EXCITE trial: attributes of the wolf motor function test in patients with subacute stroke. *Neurorehabilitation and Neural Repair*.

[24] Uswatte G, Taub E, Morris D, Vignolo M, McCulloch K (2005). Reliability and validity of the upper-extremity motor activity log-14 for measuring real-world arm use. *Stroke*.

[25] Mathiowetz V, Weber K, Volland G, Kashman N (1984). Reliability and validity of grip and pinch strength evaluations. *Journal of Hand Surgery*.

[26] Duncan PW, Lai SM, Bode RK, Perera S, DeRosa J (2003). Stroke impact scale-16: a brief assessment of physical function. *Neurology*.

[27] Brasil-Neto JP, Cohen LG, Panizza M, Nilsson J, Roth BJ, Hallett M (1992). Optimal focal transcranial magnetic activation of the human motor cortex: effects of coil orientation, shape of the induced current pulse, and stimulus intensity. *Journal of Clinical Neurophysiology*.

[28] Rossini PM, Barker AT, Berardelli A (1994). Non-invasive electrical and magnetic stimulation of the brain, spinal cord and roots: basic principles and procedures for routine clinical application. Report of an IFCN committee. *Electroencephalography and Clinical Neurophysiology*.

[29] Gowland C, Stratford P, Ward M (1993). Measuring physical impairment and disability with the Chedoke-McMaster Stroke Assessment. *Stroke*.

[30] Schulz KF, Altman DG, Moher D (2010). CONSORT 2010 statement: updated guidelines for reporting parallel group randomized trials. *Annals of Internal Medicine*.

[31] Iqbal R, Haroon A, Jabbar A, Babar N, Qureshi R (2012). What method of contact works best for recruiting participants in a study: lessons for health care researchers?. *Journal of Pakistan Medical Association*.

[32] Seniow J, Bilik M, Lesniak M, Waldowski K, Iwanski S, Czlonkowska A (2012). Transcranial magnetic stimulation combined with physiotherapy in rehabilitation of poststroke hemiparesis: a randomized, double-blind, placebo-controlled study. *Neurorehabilitation and Neural Repair*.

[33] Avenanti A, Coccia M, Ladavas E, Provinciali L, Ceravolo MG (2012). Low- frequency rTMS promotes use-dependent motor plasticity in chronic stroke: a randomized trial. *Neurology*.

[34] Conforto AB, Anjos SM, Saposnik G (2012). Transcranial magnetic stimulation in mild to severe hemiparesis early after stroke: a proof of principle and novel approach to improve motor function. *Journal of Neurology*.

[35] Maeda F, Keenan JP, Tormos JM, Topka H, Pascual-Leone A (2000). Interindividual variability of the modulatory effects of repetitive transcranial magnetic stimulation on cortical excitability. *Experimental Brain Research*.

[36] Gangitano M, Valero-Cabré A, Tormos JM, Mottaghy FM, Romero JR, Pascual-Leone Á (2002). Modulation of input-output curves by low and high frequency repetitive transcranial magnetic stimulation of the motor cortex. *Clinical Neurophysiology*.

[37] Adeyemo BO, Simis M, Macea DD, Fregni F (2012). Systematic review of parameters of stimulation, clinical trial design characteristics, and motor outcomes in non-invasive brain stimulation in stroke. *Frontiers in Psychiatry*.

